# HATL5: A Cell Surface Serine Protease Differentially Expressed in Epithelial Cancers

**DOI:** 10.1371/journal.pone.0087675

**Published:** 2014-02-03

**Authors:** Gregory S. Miller, Gina L. Zoratti, Andrew S. Murray, Christopher Bergum, Lauren M. Tanabe, Karin List

**Affiliations:** 1 Department of Pharmacology, Wayne State University School of Medicine and Barbara Ann Karmanos Cancer Institute, Detroit, Michigan; 2 Department of Oncology, Wayne State University School of Medicine and Barbara Ann Karmanos Cancer Institute, Detroit, Michigan; Vanderbilt University, United States of America

## Abstract

Over the last two decades, cell surface proteases belonging to the type II transmembrane serine protease (TTSP) family have emerged as important enzymes in the mammalian degradome, playing critical roles in epithelial biology, regulation of metabolic homeostasis, and cancer. Human airway trypsin-like protease 5 (HATL5) is one of the few family members that remains uncharacterized. Here we demonstrate that HATL5 is a catalytically active serine protease that is inhibited by the two Kunitz type serine protease inhibitors, hepatocyte growth factor activator inhibitor (HAI)-1 and 2, as well as by serpinA1. Full-length HATL5 is localized on the cell surface of cultured mammalian cells as demonstrated by confocal microscopy. HATL5 displays a relatively restricted tissue expression profile, with both transcript and protein present in the cervix, esophagus, and oral cavity. Immunohistochemical analysis revealed an expression pattern where HATL5 is localized on the cell surface of differentiated epithelial cells in the stratified squamous epithelia of all three of these tissues. Interestingly, HATL5 is significantly decreased in cervical, esophageal, and head and neck carcinomas as compared to normal tissue. Analysis of cervical and esophageal cancer tissue arrays demonstrated that the squamous epithelial cells lose their expression of HATL5 protein upon malignant transformation.

## Introduction

The type II transmembrane serine proteases are divided into four phylogenetically distinct subfamilies: the human airway trypsin-like (HAT)/differentially expressed in squamous cell carcinoma gene (DESC) subfamily, hepsin/transmembrane protease serine (hepsin/TMPRSS) subfamily, matriptase subfamily, and the corin subfamily. HATL5 belongs to the HAT/DESC subfamily together with HAT, DESC1, TMPRSS11A, and HAT-like 4 [1]–. HATL5 (HATL-5, HAT-like 5) is encoded by the TMPRSS11b gene located within a single gene cluster encompassing all the HAT/DESC genes in both mice and humans [4]. All members of the HAT/DESC subfamily are comprised of a stem region with a single sea urchin sperm protein, enteropeptidase, agrin (SEA) domain, and a C-terminal serine protease domain. There is an extensive body of literature documenting critical roles of members of the hepsin/TMPRSS, matriptase, and corin subfamilies in physiological and pathological processes. Critical roles for these TTSPs have been described in diverse areas and include epithelial development and homeostasis, iron metabolism, hearing, digestion, blood pressure regulation, as well as viral infection, inflammation, and oncogenesis [3] [5] [6]. Comparatively few studies characterizing the biochemical properties of the HAT/DESC subfamily and/or exploring their physiological functions have been published. HAT has been reported to have fibrinogenolytic activity, to modulate the urokinase receptor, and to activate protease activated receptor (PAR) 2 [7] [8] [9] [10] [11]. In addition, HAT can uncoat reovirus virions to promote infection in cell culture and cleaves the surface glycoprotein, hemagglutinin (HA), of the influenza virus [12] [13] [14]. Recently, a study employing genetic ablation of TMPRSS11A and HAT in mice demonstrated that the two proteases are dispensable for development, general health, and long-term survival in the absence of external challenges or additional genetic deficits [15].

In this study, we performed a biochemical characterization and expression analysis of HATL5. The full-length HATL5 cDNA directs the expression of a 60 kDa N-glycosylated protein that localizes to the cell surface of mammalian cells. The purified activated HATL5 serine protease domain hydrolyzes synthetic peptide substrates, and is inhibited by members of two different serine protease inhibitor families: the Kunitz-type; HAI-1, HAI-2 and aprotinin, and the serpin family member; serpinA1. HATL5 protein localization is remarkably similar in the three different tissues analyzed: cervix, esophagus, and oral mucosa. Thus, HATL5 is mainly detected on the surface of epithelial cells in these stratified squamous epithelia. During carcinogenesis, expression of the cell-surface protease is largely diminished, and in many cases, undetectable in the squamous carcinoma cells.

## Materials and Methods

### Ethics Statement

The use of human tissue paraffin arrays was approved according to the institutional guidelines by the Wayne State University Institutional Review Board Administration (#2013-43).

### Cloning and Expression of Full-length Human HATL5

Human esophageal RNA was obtained from Biochain (Newark, CA). First strand cDNA synthesis was performed with Oligo (dT) primers using a RETROscript kit according to the manufacturers’ instructions (Ambion, Life Technologies, Grand Island, NY). Gene specific primers were designed for full-length human HATL5 using the deposited sequence for *Homo sapiens* transmembrane protease, serine 11B, mRNA, GenBank#BC126195.1. The primers 5′- GCCACCATGTAC-AGGCACGGCATATC-3′ and 5′- GAGTCCAGTCTTGGATGTAATCC-3′ were used to amplify the cDNA using a high-fidelity Platinum®Taq polymerase (Invitrogen, Life Technologies, Grand Island, NY) which was then inserted into pcDNA 3.1/V5-His TOPO® TA (Invitrogen, Life Technologies, Grand Island, NY) in frame with a C-terminal HIS-tag and V-5 epitope. Constructs were verified by sequencing (ABI Prism 3730 DNA Analyzer, Invitrogen, Life Technologies, Grand Island, NY). Transfection of HEK293 and COS-7 cells (ATCC, Manassas, VA) was performed using Lipofectamine 2000 according to the manufacturer’s instructions (Invitrogen, Life Technologies, Grand Island, NY). Cells were cultured in Dulbecco’s modified Eagle’s media (Gibco, Life Technologies, Grand Island, NY) supplemented with 10% fetal bovine serum (Atlanta Biologicals, Lawrenceville, GA). Transfection was performed with 4.0 µg full-length HATL5-containing plasmid DNA. Cells were lysed using RIPA buffer: 150 mM NaCl, 50 mM Tris/HCl, pH 7.4, 0.1% SDS, 1% NP-40, and protease inhibitor cocktail (Sigma-Aldrich, St. Louis, MO) and cleared by centrifugation at 12,000×g at 4C°. Protein concentrations were determined using a Pierce BCA Protein Assay Kit (Thermo Scientific, Waltham, MA). Protein samples were separated by SDS-PAGE using 4–12% NuPAGE Bis-Tris gels (Life Technologies, Grand Island, NY) under reducing conditions and analyzed by western blotting using a primary V-5 mouse monoclonal antibody (Invitrogen, Life Technologies, Grand Island, NY) and a goat anti-mouse secondary alkaline phosphatase-conjugated antibody (Millipore, Billerica, MA).

### Cloning and Expression of Human HATL5 Serine Protease Domain Pro-form

The human HATL5 serine protease (SP) domain was cloned into the pSecTag2a plasmid (Life Technologies, Grand Island, NY) for expression and analysis in mammalian cell culture. HATL5 serine protease domain sequence from the human full-length HATL5-V5-His plasmid was PCR amplified using the following primers: 5′-CCAAGGATCCATGTTGTGGGAGACAAGTAGCCAAC-3′ and 5′-CCAAGCGGCCGCGAGTCCAGTCTTGGATGTAATCC-3′. The resulting PCR fragment was cloned into the pSecTag2a vector between the BamHI and NotI sites using standard techniques. Transfection of CHO cells (ATCC, Manassas, VA), protein extraction, and western blot analysis was performed as described above. A mouse monoclonal anti-Myc antibody (Invitrogen, Life Technologies, Grand Island, NY) was used for detection by western blotting.

### Cloning and Expression of the HATL5 Active Serine Protease Domain in *Pichia pastoris*

The human HATL5 active serine protease domain was produced in yeast using a Pichia Expression Kit (Invitrogen, Life Technologies, Grand Island, NY). For cloning into the pPIC9 vector, HATL5 serine protease sequence from the pcDNA 3.1 V5/HIS/TOPO human-HATL5 plasmid was mutated to remove an XhoI site using the Agilent Quick-Change II™ site directed mutagenesis kit (Agilent Technologies, Inc. Santa Clara, CA). Primers for mutagenesis were: 5′-gccaggtgtctatactcgggtgacttcttatcgcaattgg-3′ and 5′-ccaattgcgataagaagtcacccgagtatagacacctggc-3′. Mutagenesis resulted in a single synonymous point mutation, thus reproducing the native amino acid sequence. Following mutagenesis, human HATL5 serine protease domain was amplified and cloned into the pPIC9 vector at the XhoI and NotI sites, using the primers 5′-tctctcgagaaaagaattgtgaatggaaaaagctccc-3′ and 5′-attcgcggccgctcagagtccagtcttgg-3′. Cloning resulted in HATL5 activated serine protease domain sequence (Ile-Val-Asn) being inserted immediately following a Leu-Glu-Lys-Arg KEX2 cleavage site encoded by the vector. The Leu-Glu-Lys-Arg-Ile-Val-Asn is cleaved between Arg and Ile by the yeast protease KEX2 which is a transmembrane protease located in the Golgi, rendering a secreted activated HATL5 serine protease domain. Expression of matriptase active serine protease domain in *Pichia pastoris* was performed in parallel as described [19]. Transformation was performed in TOP-10 competent cells (Invitrogen, Life Technologies, Grand Island, NY) and pPIC9-HATL5 positive clones were isolated and amplified using standard techniques. For transformation of *Pichia pastoris*, 40 µg of pPIC9-human-HATL5, pPIC9-matriptase, pPIC9 empty vector was digested with SalI, and purified by phenol-chloroform extraction. Electroporation of linearized plasmid into the GS115 yeast strain (Invitrogen, Life Technologies, Grand Island, NY) was performed at 1.5 kV using 0.2 cm cuvettes (BioRad, Hercules, CA) in a BTX-Transporator Plus **(**Harvard Apparatus Holliston, MA). The expression of recombinant proteases in the conditioned media from individual yeast clones was analyzed by SDS-PAGE and western blotting using a rabbit anti-TMPRSS11b (Abcam, Cambridge, MA) or a rabbit anti-matriptase antibody (Bethyl Laboratories, Montgomery, TX). HATL5 and matriptase proteins were purified by anion-exchange chromatography using a HiTrap Q HP column (GE Healthcare, Pittsburgh, PA) as described [16].

### Chromogenic Proteinase Assays

The assays were performed in 96-well plates in a total reaction volume of 100 µL using 50 mM Tris-HCl pH 8.0, 150 mM NaCl, 0.01% Tween-20, 0.01% BSA for dilution of all samples. 5 nM purified active recombinant HATL5 or matriptase serine protease domain was incubated at 37°C for 60 min with 100 µm of the synthetic peptide L-1720 Suc-Ala-Ala-Pro-Arg-pNA (Bachem, Bubendorf, Switzerland) in the absence or presence (inhibitor and substrate added concomitantly) of HAI-1 (60 nM) (R&D, Minneapolis, MN), HAI-2 (40 nM) (R&D, Minneapolis, MN), aprotinin (2 µm), leupeptin (20 µm), benzamidine (2 mM), or serpinA1 (60 nM) (all from Thermo Scientific, Waltham, MA). Changes in absorbance at 405 nm were monitored using a Magellan NanoQuant Infinite M200 Pro plate reader (Tecan US, Inc., Morrisville, NC).

### Deglycosylation of HATL5

Proteins in lysates or conditioned media prepared as indicated above were deglycosylated using an Enzymatic Protein Deglycosylation Kit according to the manufacturer’s instructions (Sigma, St. Louis, MO).

### Immunocytochemistry

Cell-surface imaging was performed using HEK293 or COS-7 cells transfected with human full-length HATL5-V5. Cells were seeded on coverslips coated with rat type-2 collagen (BD Biosciences, Franklin Lakes, NJ) and allowed to adhere and grow for 36 h, at which point, media was removed and cells were fixed in 4% paraformaldehyde in PBS for 30 min at room temperature. HATL5-V5 was detected using a monoclonal anti-V5 antibody (Invitrogen, Life Technologies, Grand Island, NY) or an isotype control antibody (Sigma, St. Louis, MO) and a secondary AlexaFlour-568-conjugated goat-anti-mouse antibody (Invitrogen, Life Technologies, Grand Island, NY). Cell staining was imaged by confocal microscopy using a Leica TCS SP2 confocal microscope (Leica, Buffalo Grove, IL).

### Bioinformatics Analysis

The Oncomine microarray database (http://www.oncomine.org) [17] was used to perform a meta-analysis of the expression of human *TMPRSS11b* across four studies of the transcriptome in carcinomas of the cervix, esophagus, and head and neck, as compared with normal control tissues (Table S1).

### Tissue Samples and Immunohistochemistry

The “CR802”, “ES482”, and “T271” cervical, esophageal, and oral cancer tissue arrays, including normal or cancer adjacent normal tissue, were obtained from US Biomax, Inc. (Rockville, MD).

Tissue arrays were deparaffinized with xylene and hydrated with graded ethanol solutions. Antigen retrieval was performed using citrate buffer, reduced pH (Bethyl Laboratories, Montgomery, TX). The arrays were blocked with 2.5% normal horse serum (Vector Laboratories, Burlingame, CA) in PBS, and immunostained overnight at 4°C with 4 µg/ml rabbit anti human TMPRSS11b/HATL5 (Sigma, St. Louis, MO). As a negative control, non-immune rabbit IgG (4 µg/ml)(Millipore, Billerica, MA) was used. Bound antibodies were visualized using biotin-conjugated anti-rabbit (Vector Laboratories, Burlingame, CA) secondary antibodies, and a Vectastain ABC kit (Vector Laboratories, Burlingame, CA). The 3,3′-diaminobenzidine was used as the substrate (Sigma, St. Louis, MO) and arrays were counterstained with hematoxylin. All microscopic images were acquired on a Zeiss Scope A.1 using digital imaging.

### Evaluation of Staining Intensities and Statistical Analysis

Assessment of staining intensities of esophageal and cervical tissue samples was performed by an investigator unaware of sample identity. Scores were assigned on the basis of the intensity and extent of epithelial staining in 20x microscopic fields using an arbitrary scale from zero to three where 0 = no positive staining in epithelial cells detected, 1 = some epithelial cells stained weakly, 2 = some epithelial cells stained moderately or the majority of epithelial cells stained weakly, 3 = some epithelial cells stained strongly or the majority of epithelial cells stained moderately. Two-tailed χ2 analysis was used to determine statistical significance of differences in the frequency of HATL5 staining between groups. Statistical significance of the difference in HATL5 staining intensity between groups was determined by the two-tailed Mann–U Whitney test.

## Results

### Amino Acid Sequence and Domain Structure of HATL5

**Figure 1:**
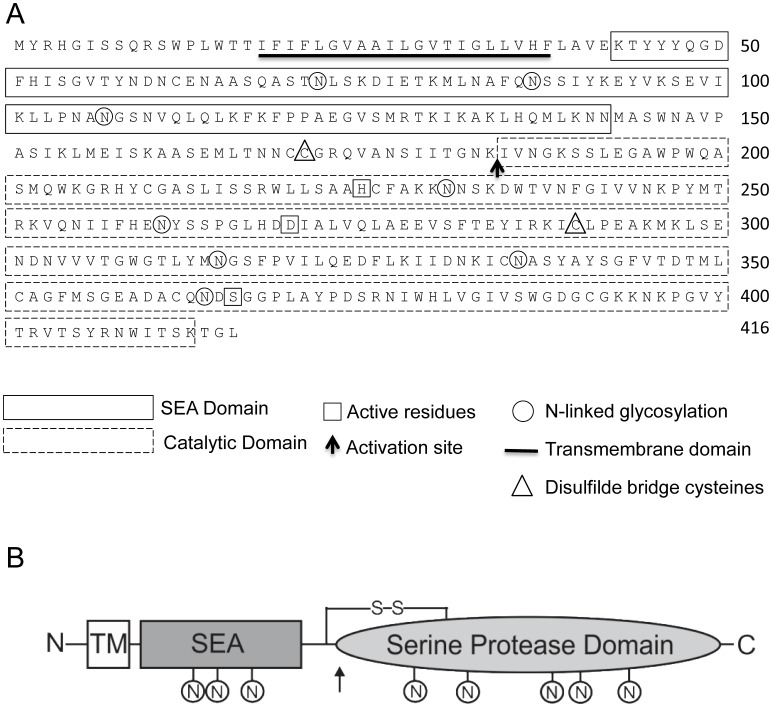
Amino acid sequence, and predicted domain architecture of human HATL5. (**A**) Amino acid sequence of human HATL5 (UniProtKB/Swiss-Prot: Q86T26.3). Amino acid residues encoding the transmembrane domain are underlined. The region with homology to sea urchin sperm protein, enteropeptidase, agrin (SEA) domains is indicated by a solid line box and the trypsin-like serine protease domain is indicated with a dashed line box. The catalytic residues His^225^ (H), Asp^270^ (D), and Ser^366^ (S) are indicated with squares. Potential N-glycosylation sites are indicated with circles. The activation cleavage site is indicated with an arrow, and the two cysteine residues predicted to form a disulfide bridge linking the stem region to the serine protease domain upon activation cleavage are indicated with triangles. (**B**) Schematic representation of the predicted domain architecture of HATL5. TM = Transmembrane domain, SEA = Sea urchin sperm protein, Enteropeptidase, Agrin domain, N indicates predicted N-glycosylation sites, the activation cleavage site is indicated with an arrow, and S-S represents the disulfide bridge linking the SEA and serine protease domains [31].

Analysis of the amino acid sequence of the putative human HATL5 protease revealed eight potential N-glycosylation sites; five of which are located in the serine protease domain (Fig. 1A and B). The catalytic domain of mouse HATL5 contains the essential serine protease catalytic triad residues, H^225^, D^270^, S^366^, and the substrate binding pocket residues, D^360^, S^386^ and G^388^. The SWG motif in the catalytic domain is conserved in all members of the HAT/DESC subfamily and is predicted to be located at the top of the substrate binding pocket, positioning the scissile bond of the substrate in the correct orientation (S^386^WG in HATL5). Proteolytic activation of HATL5 is predicted to occur within a motif (K^184^IVNG) at the junction of the pro- and catalytic domains. These findings suggest that the *TMPRSS11b* gene likely encodes a functional serine protease.

### HATL5 is a 60 kDa Glycosylated Protein

**Figure 2:**
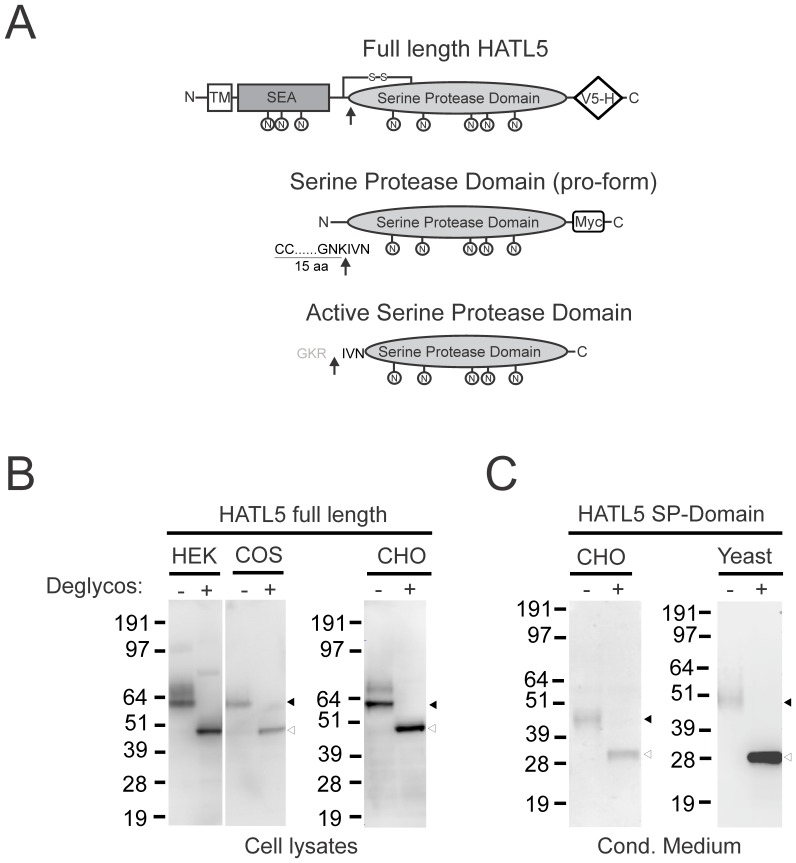
HATL5 is a 60-kDa glycoprotein. (**A**) Schematic representation of the three different recombinant HATL5 proteins generated for this study. V5-H = V5-His epitope tag (**B**) Whole cell protein lysates from HEK293, COS-7, or CHO cells expressing full-length V5-His tagged human HATL5. (**C**) Conditioned media from CHO cells expressing myc-tagged HATL5 serine protease domain or conditioned media from *Pichia pastoris* expressing cleaved, active HATL5 serine protease were analyzed. (**B and C**) Proteins were separated by SDS-PAGE and analyzed by western blotting using anti-V5, anti-myc or anti-HATL5 antibodies as indicated. Lanes with protein extracts treated with deglycosylation enzymes prior to SDS-PAGE are indicated (+), and untreated extracts (−). The black arrowheads indicate the position of the glycosylated forms of HATL5, and the open arrowheads indicate the position of the deglycosylated forms.

To characterize the HATL5 protein encoded by the *TMRSS11b* gene, the full-length human cDNA was generated by high-fidelity PCR using a human esophagus cDNA library. The cDNA was sequenced, confirmed to be identical to the predicted cDNA sequence, and was inserted into a mammalian expression plasmid that furnished the expressed protein with a V5-His epitope tag at the C- terminus (Fig. 2A, top). In addition, a mammalian expression vector containing the pro-form of the serine protease domain, including 15 amino acids upstream from the activation site, and a C-terminal Myc-tag was generated (Figure 2A, middle) as well as a *Pichia pastoris* vector for secretion of the cleaved active form of the serine protease domain (Figure 2A, bottom). After transfection of the full-length HATL5 vector in HEK293, COS-7, or CHO cells, respectively, the cell lysates were analyzed by SDS-PAGE and western blot. In all three cell lines, a protein product of approximately 60 kDa was detected (Fig. 2B). This apparent molecular mass is significantly higher than the predicted molecular mass of 46 kDa, suggesting that HATL5 is subject to post-translational modifications. To examine whether the expressed HATL5 may be post-translationally modified by glycosylation, protein extracts from transfected HEK293, COS-7, or CHO cells were subjected to enzymatic deglycosylation prior to western blot analysis. Deglycosylation of full-length HATL5 led to the emergence of a ∼48 kDa species that was present in extracts from all three cell lines (Fig. 2B). Taking into consideration that the V5-His tag adds approximately 5 kDa to the recombinant protein, the deglycosylated form of full-length HATL5 has an apparent molecular mass very close to the predicted molecular mass. Five of the eight potential N-glycosylation sites are located in the serine protease domain. To determine whether the serine protease domain of HATL5 is glycosylated, the serine protease pro-form secreted by transfected CHO cells was treated with deglycosylation enzymes and analyzed by western blotting. Prior to deglycosylation this form had an apparent molecular mass of 45 kDa (Fig. 2C, left). The predicted molecular mass of this secreted form of HATL5 is 30 kDa, including the myc-tag. Upon deglycosylation, a reduction in the apparent molecular mass, to a 30 kDa form, was observed. A similar observation was made upon deglycosylation of the untagged cleaved active HATL5 serine protease domain secreted by transfected *Pichia pastoris* (predicted molecular mass = 26 kDa) that resulted in a form with an apparent molecular mass of ∼28 kDa (Fig. 2C, right). Taken together, this analysis demonstrates that human HATL5 is a 60-kDa glycoprotein and that one or more of the five potential *N*-linked glycosylation sites in the serine protease domain are utilized.

### HATL5 is a Catalytically Active Protease

**Figure 3:**
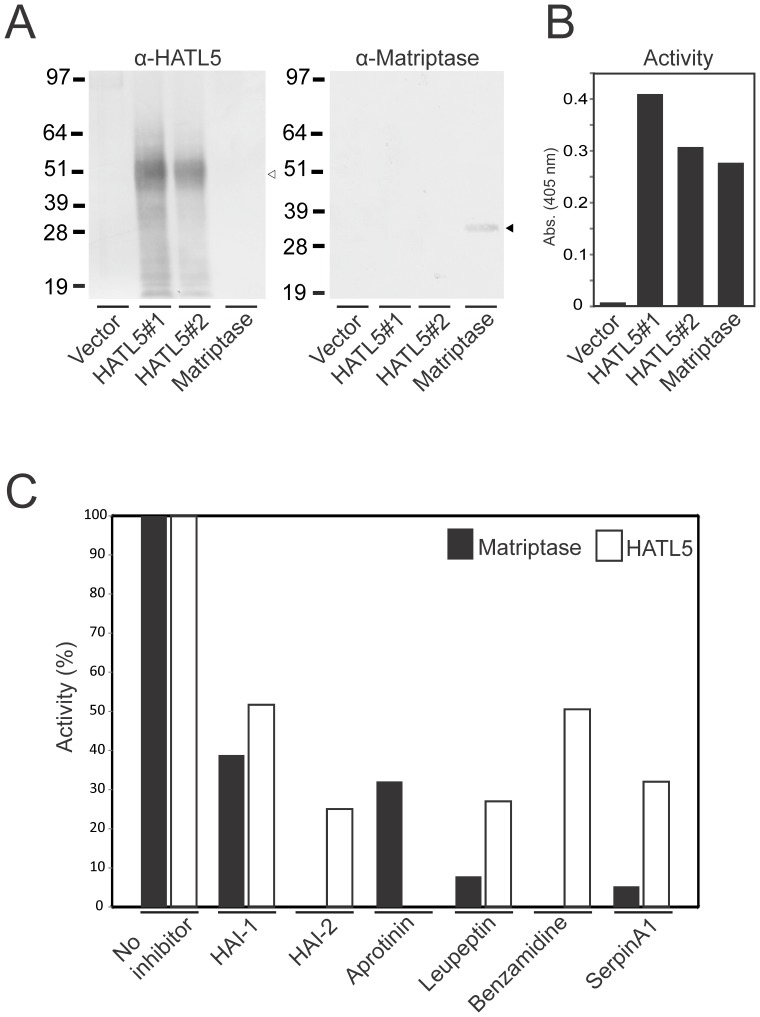
Analysis of the enzymatic activity of human HATL5. (**A**) Conditioned media samples from *Pichia pastoris* clones transfected with either the expression vector without protease insert (vector), with HATL5 serine protease domain cDNA (HATL5 #1 and #2) or matriptase serine protease domain cDNA were analyzed by western blotting using an anti-HATL5 antibody (left panel) or an anti-matriptase antibody (right panel). The positions of HATL5 (open arrow head) and matriptase serine protease domain (black arrow head) are indicated. (**B**) Samples from the conditioned media described in (A) were incubated at 37°C for 60 min with the synthetic chromogenic peptide Suc-Ala-Ala-Pro-Arg-pNA (100 µm) and the absorbance at 405 nm was recorded (**C**) 5 nM purified active recombinant HATL5 (white bars) or matriptase (black bars) serine protease domain was incubated at 37°C for 60 min with the synthetic chromogenic peptide MeOSuc-Glu-Val-Lys-Met-pNA (100 µm) in the absence or presence (inhibitor and substrate added concomitantly) of HAI-1 (60 nM), HAI-2 (40 nM), aprotinin (2 µm), leupeptin (20 µm), benzamidine (2 mM), or serpinA1 (60 nM). Enzyme activities for each enzyme are depicted relative to activity when no inhibitor was added.

To investigate the enzymatic properties of HATL5, the serine protease domain, including the activation cleavage site and 15 additional amino acids upstream from the cleavage site, was expressed in CHO cells (Fig. 2 middle). The HATL5 protein was secreted into the media as a single chain pro-enzyme. A similar approach previously employed for matriptase-3 led to a secreted protein capable of undergoing auto-activation as evidenced by a slight increase in electrophoretic mobility and by its acquired proteolytic activity [18]. The secreted form of HATL5 does not, however, appear to have auto-catalytic properties since incubation under various different conditions (buffer composition, temperature, and time) did not yield any detectable mobility shift or catalytic activity (data not shown). In order to express HATL5 and mediate proteolytic cleavage at the activation site, the *Pichia pastoris* expression system was employed utilizing the intracellular yeast protease, KEX2. The KEX2 transmembrane serine protease belongs to the subtilisin-like pro-protein convertase family with specificity for cleavage after paired basic amino acids and is localized in the late Golgi compartment. By cloning the HATL5 serine protease domain into the PIC9 vector with the HATL5 active serine protease domain sequence (^185^IVNG) immediately following the LGKR KEX2 cleavage site encoded by the vector, a novel fusion cleavage site was generated (Fig. 2, bottom). The LGKRIVNG sequence is cleaved between Arg (R) and Ile (I) by KEX2, rendering a secreted active HATL5 serine protease domain. Expression of matriptase active serine protease domain in *Pichia pastoris* was performed in parallel as described [19]. The presence of HATL5 serine protease in conditioned media from *Pichia pastoris* clones transfected with the PIC9-HATL5 vector was confirmed by western blotting using an anti-HATL5 antibody (Fig. 3A, left). No signal was detected in conditioned media from clones transfected with the empty PIC9 vector or the PIC9-matriptase vector. Similarly, the anti-matriptase antibody detected the secreted matriptase serine protease domain in conditioned media from cells transfected with the PIC9-matriptase vector (Fig. 3A, right) and not from clones transfected with the PIC9-HATL5 expression vector or the PIC9 vector without protease insert. The catalytic activity of HATL5 and matriptase, respectively, was confirmed using the serine protease chromogenic peptidolytic substrate Suc-Ala-Ala-Pro-Arg-pNA (Fig. 3B). Conditioned media from cells transfected with empty PIC9 vector was included as a negative control to ensure the absence of interfering secreted yeast proteases.

The effect of various serine protease inhibitors on the hydrolytic activity of the purified HATL5 serine protease domain towards Suc-Ala-Ala-Pro-Arg-pNA was also analyzed, again using matriptase as a well-characterized reference TTSP (Fig. 3C). HATL5 activity was reduced by the generic molecule inhibitors of serine proteases, leupeptin and benzamidine. Complete inhibition was achieved with the globular polypeptide, Kunitz type serine protease inhibitor, aprotinin (bovine pancreatic trypsin inhibitor). The macromolecular Kunitz type serine protease inhibitors, hepatocyte growth factor activator inhibitor (HAI)-1 and 2, have previously been shown to inhibit several members of the TTSP family including matriptase, matriptase-2, HAT, hepsin and TMPRSS13 [20] [21] [22] [23] [24] [25] [26] [27]. HAI-1 and HAI-2 inhibited the activity of HATL5 by 50% and 75%, respectively. In addition, HATL5 was inhibited by a member of the serpin superfamily, serpinA1 (α_1_-1-antitrypsin).

### HATL5 is Localized on the Cell Surface of Cultured Cells and in Stratified Squamous Epithelia

**Figure 4:**
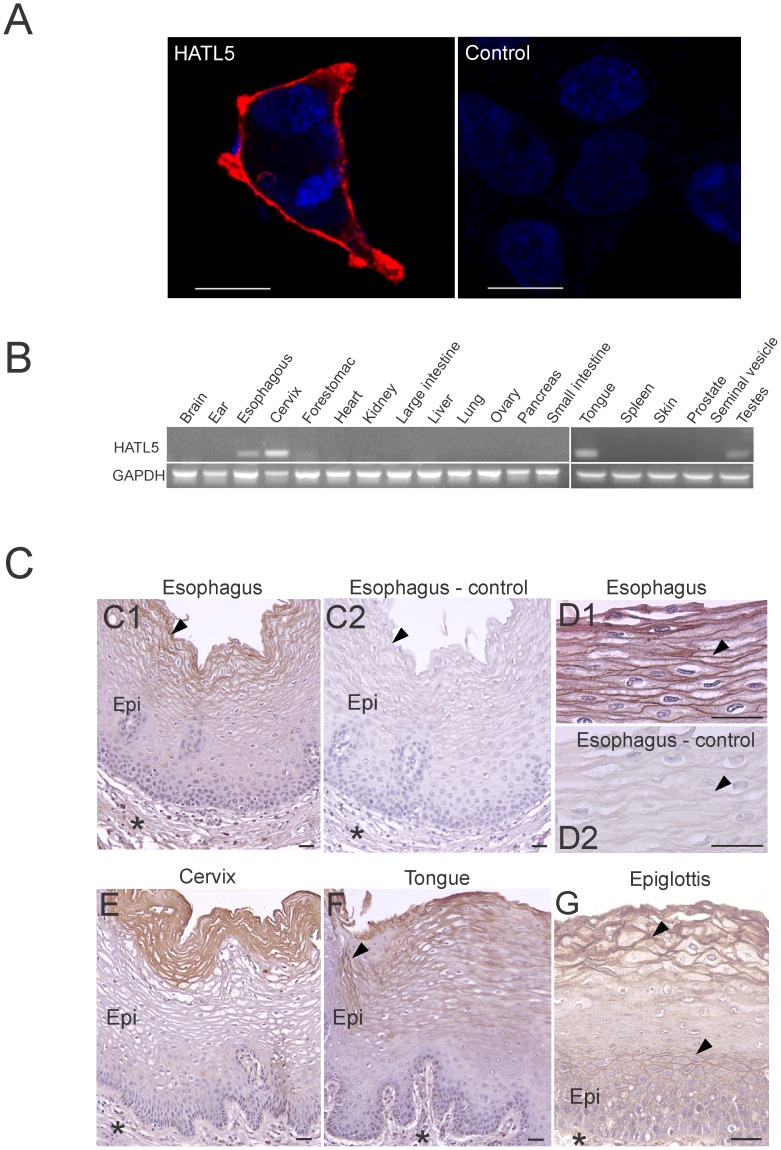
HATL5 expression and localization. (**A**) Micrographs of fluorescent confocal analysis showing cell surface expression in representative examples of HEK293 cells transiently transfected with a human full-length HATL5-V5 expression plasmid. Nonpermeabilized cells were incubated with an anti-V5 antibody (left panel) or a control non-immune antibody (right panel), followed by incubation with AlexaFluor 568-labeled secondary antibodies and Hoechst staining to visualize nuclei (blue; both panels). The cells were visualized by confocal fluorescence microscopy at 543 nm (AlexaFluor 568) and 405 nm excitation wavelengths (Hoechst). Merged images obtained at the two excitation wavelengths are shown. Size bars are 100 µm. (**B**) Expression of HATL5 (upper panel) or GAPDH (lower panel) message by RT-PCR analysis of mRNA extracted from whole mouse organs. (C) Immunohistochemical analysis of HATL5 expression in normal human tissues. HATL5 protein was detected with a rabbit-anti HATL5 antibody in esophageal musoca (C1, D1), cervical mucosa (E), oral mucosa (tongue) (F) and epiglottis (part of the supraglottic larynx) (G). Primary antibodies were substituted with non-immune rabbit IgG in serial sections of all samples and no significant staining was observed (arrowheads in C2, D2 and not shown). Strong epithelial staining (arrowheads) is detected in apical, squamous epithelial cells in normal esophagus (C1, D1), normal cervix (E), and tongue (F) with no significant staining in the mesenchymal compartment (indicated with asterisks). At high magnification, HATL5 protein is clearly localized on the cell surface of apical epithelial cells (arrow head in D1). In the epiglottis (G) staining is observed in squamous suprabasal epithelium cells in addition to apical cells. Epi = normal epithelium. Size bars all panels; 50 µm.

Analysis of the HATL5 protein sequence revealed the presence of a single transmembrane domain, indicating that the HATL5 protein, similar to previously characterized members of the TTSP family, may be located on the cell surface. To investigate the subcellular localization of HATL5, HEK293 cells expressing the V5-tagged full-length protease were analyzed by fluorescent immunocytochemistry. Nonpermeabilized transfected cells stained with a primary anti-V5 antibody and an AlexaFluor 568-conjugated secondary antibody were analyzed by confocal fluorescence microscopy. Transfected HEK293 cells displayed an intense red fluorescent signal that was confined to the plasma membrane (Fig. 4A, left panel). When primary antibodies were substituted with non-immune IgG, no detectable staining was observed (Fig. 4A, right panel). A similar membrane staining was observed in a parallel experiment using transfected COS-7 cells (data not shown).

To determine the expression of HATL5 in tissues, RT-PCR analysis was performed on total RNA extracted from a series of adult mouse tissues (Fig. 4B). This analysis revealed that HATL5 displays a relatively restricted tissue expression profile, with mRNA detected in 4 out of the 19 tissues analyzed: esophagus, cervix, tongue, and testes. A common feature between the esophagus, cervix, and tongue is that the mucosal linings of these tissues all contain stratified squamous epithelium. This prompted us to examine the distribution and cellular localization of HATL5 protein in these tissues by immunohistochemistry (IHC). Representative sections of normal esophageal, cervical, and head and neck (tongue and epiglottis) mucosa are shown in Figure 4C. Using serial sections, slides were probed with a rabbit anti-HATL5 protein or used as negative controls where the primary antibody was substituted with non-immune rabbit IgG (Fig. 4C and data not shown). In all three tissue types a strong epithelial staining is detected in the apical squamous cells with no significant staining in the submucosal or mesenchymal compartments. HATL5 protein is primarily localized on the cell surfaces of the larger squamous cells. This cell surface localization is in agreement with the expected distribution of the predicted membrane anchored topology of HATL5. No significant staining was detected in the small, cuboidal and highly mitotic cells in the basal layer.

Taken together, these data demonstrate that the *TMPRSS11b* gene directs the synthesis of a transmembrane glycoprotein that is expressed on the cell surface of cultured cells and in intact tissues. The endogenous HATL5 protein displays a pericelluar localization in differentiating epithelial cells in stratified squamous epithelia from diverse tissues.

### Loss of HATL5 Expression in Squamous Cell Carcinomas

**Figure 5:**
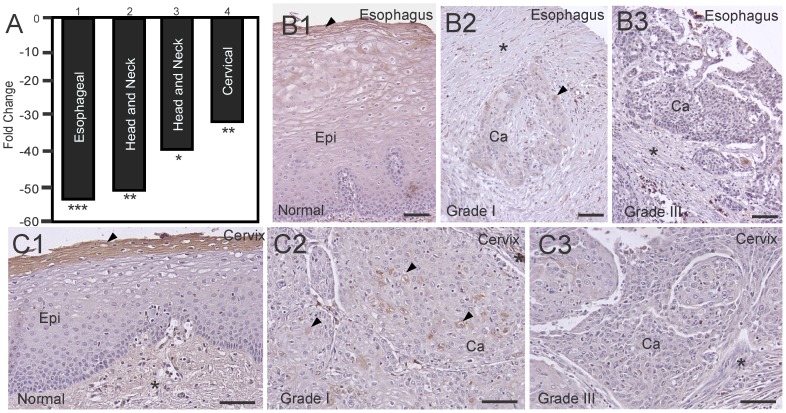
Decreased expression of HATL5 mRNA and protein in human carcinomas. (**A**) TMPRSS11b, encoding HATL5 in four (1–4) gene expression array studies of human carcinomas (see Table S1). Data are expressed as fold change in HATL5 mRNA relative to corresponding normal tissue. *P<4·10^−5^, **P<4·10^−10^, ***P<2·10^−23^. (**B**) IHC detection of HATL5 protein in esophageal tissue (B1, B2, B3) and cervical tissue sections (C1, C2, C3). Primary antibodies were substituted with non-immune rabbit IgG in serial section of all samples and no significant staining was observed (not shown). Strong epithelial staining (arrow heads) is detected in normal esophagus (B1) and normal cervix (C1). With carcinoma progression (Grade I) the HATL5 staining was weaker and more diffuse with only a few moderately stained carcinoma cells (arrowheads in B2 and C2) and in high grade poorly differentiated tumors (Grade III) the staining intensity was very low or below the detection limit of this assay (B3 and C3). Epi = normal epithelium, Ca = carcinoma cells. Size bars all panels; 50 µm.

**Figure 6:**
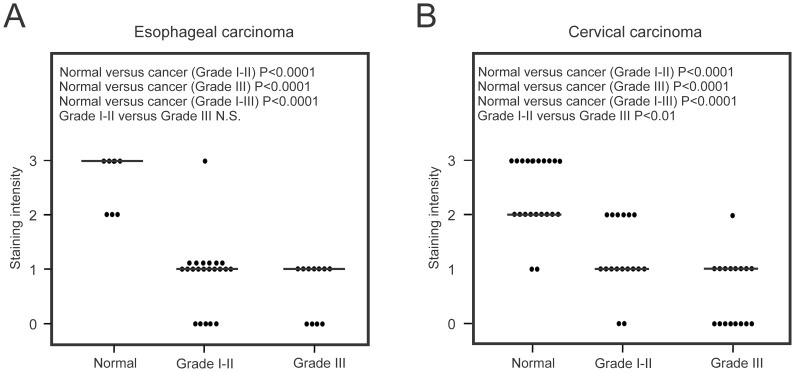
Decreased HATL5 expression in esophageal and cervical carcinomas. Scatterplots illustrating the intensity of IHC staining of HATL5 in tissue array sections. Horizontal bars represent median values. Staining intensities were determined as described in Material and Methods. (**A**) In esophageal squamous cell carcinomas, a highly significant decrease in staining intensity (P<0.0001) was observed between normal tissues and each of the two groups of cancer tissues; grades I-II and grade III, respectively. (**B**) In cervical squamous cell carcinoma, a highly significant decrease in staining intensity (P<0.0001) was observed between normal tissues and each of the two groups of cancer tissues divided according to grade I-II, and grade III, respectively. Furthermore, the HATL5 staining intensity was significantly lower in the high grade (grade III) group, as compared to the grade I-II group (P<0.01).

Our interest in cell-surface proteases that are differentially expressed and functionally important in carcinogenesis prompted us to investigate the expression profiles of HATL5 in human cancers. *In silico* data mining using the Oncomine™ microarray database revealed a significant decrease in the overall abundance of HATL5 transcript in a meta-analysis of four published gene expression arrays studies including carcinoma of the esophagus, head and neck, and cervix (Fig. 5A and. Table S1). To investigate whether HATL5 protein is differentially expressed in carcinomas, we performed tissue array IHC analysis in two different human squamous cell carcinoma types. HATL5 expression was assessed by IHC analysis of human esophageal squamous cell carcinomas and cervical squamous cell carcinomas as compared to their respective tumor-adjacent and/or normal tissues. For esophageal cancer, 25 squamous cell carcinomas with grades ranging from I to III (Grade I; *n* = 7, Grade II; *n* = 13, Grade III; *n* = 5) were analyzed. Normal or tumor-adjacent tissues were included on the same array slides (*n* = 7). Serial sections were incubated with a rabbit anti-HATL5 antibody or a non-immune rabbit IgG (negative control) (Figure 5 and data not shown). HATL5 was predominantly expressed on the cell surface in normal apical, squamous epithelial cells (Fig. 5 B1). During squamous cell carcinoma progression, the epithelium gradually loses its squamous appearance and the epithelia derived carcinoma cells become smaller and abnormal in appearance. In well and moderately differentiated esophageal carcinomas (Grades I and II) HATL5 expression was weak and diffuse with few cells displaying cytoplasmic staining (Fig. 5 B2). In high*-*grade, poorly differentiated esophageal carcinoma, HATL5 predominantly appeared as very weak diffuse staining or rendered undetectable (Fig. 5 B3). No nuclear staining was observed in either normal or cancer tissue. On control slides, no staining was observed when substituting the HATL5 antibody with non-immune, species-matched IgG (data not shown.). The overall comparison of groups showed a highly significant decrease in HATL5 expression in both Grade I-II (P<0.0001) and Grade III (P<0.0001) tumor samples as compared to normal and tumor adjacent samples (Fig. 6A).

For cervical squamous cell carcinomas, 38 cervical cancer samples with grades ranging from I to III (Grade I; *n* = 4, Grade II; *n* = 17, Grade III; *n* = 17) were analyzed, and compared to 21 normal or tumor-adjacent tissues. Serial sections were incubated with either a rabbit anti-HATL5 antibody or a non-immune rabbit IgG (negative control). HATL5 was predominantly expressed on the cell surface in normal apical, squamous epithelial cells (Fig. 5 C1). In well and moderately differentiated carcinomas (Grades I and II) HATL5 expression was weak and diffuse with few cells displaying cell surface staining and others displaying cytoplasmic staining (Fig. 5 C2). In high*-*grade, poorly differentiated cervical carcinoma, HATL5 predominantly appeared as very weak diffuse staining and in many cases no detectable staining was observed (Fig. 5 C3). No nuclear staining was observed in either normal or cancer tissue. On control slides, no staining was observed when substituting the HATL5 antibody with non-immune, species-matched IgG (data not shown). The frequency of tumor samples having no detectable HATL5 protein, 12/38 (32%), was significantly lower as compared to normal samples, 0/21 (0%) (p = 0.01). The overall comparison of groups showed a highly significant decrease in HATL5 expression in both Grade I-II (P<0.0001) and Grade III (P<0.0001) tumor samples as compared to normal and tumor adjacent samples. Furthermore, higher grade tumors (Grade III) displayed significantly less overall HATL5 expression than lower grade tumors (Grade I-II), (p<0.01), indicating an inverse correlation between HATL5 expression and carcinoma progression (Fig. 6B).

## Discussion

As an ongoing effort to characterize the members of the TTSP family, we performed biochemical and expression analysis of the HAT/DESC subfamily member, HATL5. The bioinformatics analysis, followed by direct molecular and biochemical analysis presented in this paper, provide strong evidence that the human *TMPRSS11b* gene encodes a functional TTSP. The HATL5 amino acid sequence contains both a transmembrane domain/signal anchor motif and a serine protease domain with the conserved catalytic amino acid triad and substrate binding pocket residues. The expression of HATL5 cDNA in mammalian cells led to the production of a recombinant glycoprotein with one or more of the predicted five N-glycosylation sites in the serine protease domain being utilized. The recombinant soluble pro-form of the HATL5 serine protease domain had no detectable catalytic activity and did not appear to undergo auto-activation under the various different conditions applied. A previously described analysis of another HAT/DESC1 subfamily member, DESC1, rendered similar results. Expression of the recombinant DESC1 protease domain led to the generation of a zymogen that required cleavage by an exogenously added activating protease for conversion into a functionally active protease [28]. It cannot be ruled out that lack of membrane anchorage in the recombinant truncated forms of HATL5 or DESC1 may hinder auto-activation. However, similar secreted forms of proteases belonging to the matriptase subfamily, matriptase, matriptase-2 and matriptase-3, are all capable of auto-activation [18] [29] [30] [31]. Therefore, it is plausible that the ability of zymogens to auto-activate may vary between subclasses.

The activities of most TTSPs are regulated by endogenous protease inhibitors. Members of several classes of inhibitors have been shown to inhibit various TTSPs, including serpins and Kunitz-type inhibitors, *in vitro* [32] [33]. Using genetic approaches it has been demonstrated that both HAI-1 and HAI-2 are critical physiological inhibitors of matriptase [25] [26]. HATL5 is most effectively inhibited by the Kunitz-type inhibitor aprotinin/bovine pancreatic trypsin inhibitor. Partial inhibition was observed with HAI-1, HAI-2, and serpinA1/α_1_-antitrypsin. It remains to be elucidated whether regulation of HATL5 by one or more of these inhibitors occurs *in vivo*. Consistent with its structural characteristics, confocal immunofluorescence analysis of cultured mammalian cells confirmed that HATL5 protein is expressed on the cell surface. This cell surface expression was further confirmed in tissues (see below). The restricted expression profile of HATL5 mRNA in cervix, esophagus, tongue, and testes is in accordance with findings described by Sales and colleagues [15]. In addition to these four tissues, they detected HATL5 message in the trachea of both mice and humans. Interestingly, HAT/DESC proteases are coordinately expressed on the message level in both humans and mice, suggesting a level of functional redundancy [15]. It is unknown, however, whether HAT/DESC proteases are coordinately expressed on the protein level as well, and whether they are present in the same tissue compartments and cell types. Expression analysis of human tissues revealed a similar localization of HATL5 protein in the squamous stratified epithelia of the cervix, esophagus, and oral cavity. HATL5 was consistently expressed in the upper apical layers harboring the most differentiated epithelial cells and was clearly confined to the cell surface in healthy epithelia. Interestingly, in squamous cell carcinoma of the same tissues, a significant decrease and, in many cases, a complete loss of HATL5 was observed in advanced cancer. The localization of HATL5 protein in normal stratified squamous epithelia and the loss of the protease in cancer provide valuable information that may give clues to the function(s) of this protease in normal epithelial biology and carcinogenesis.

In stratified squamous epithelia, a critical balance between cell proliferation, differentiation, and death must be maintained in order for these tissues to fulfill their function. The stratified squamous epithelium acts as an effective barrier against the influx of luminal contents, including pathogens. The multiple layers also make these specialized epithelia capable of withstanding abrasive stress. The proliferating epithelial cells reside in the basal layer and cells continuously move through a complex differentiation program to generate basolateral and apical cells that are finally sloughed off. Tight junctions (TJs) separate the apical and basolateral cell surfaces to establish cell polarity and barrier function. Extracelluar proteases are known to be involved in differentiation and proper function of stratified squamous epithelia. The TTSP, matriptase, is critical for terminal differentiation, function, and TJ formation in the epidermis and oral epithelium [34] [35]. Given the expression of HATL5 in the non-proliferating suprabasal/apical layers coupled with the observation that HATL5 is lost during the dedifferentiation of epithelial cells, a hallmark of squamous cell carcinogenesis, it will be important to explore whether the observed link between loss of HATL5 and squamous cell carcinoma progression is correlational or causal.

Currently, proteolytic substrates have not been identified for DESC1, TMPRSS11A, and HAT-like-4. A screening of proteins that have previously been identified as *in vivo* and/or *in vitro* substrates for other TTSPs including pro-uPA, pro-HGF, and pro-prostasin, were not activated by HATL5 in cell-free or cell-containing experimental set-ups (unpublished data). An important task ahead is to identify substrates for HATL5 in stratified squamous epithelia cells and other cells/tissues expressing the protease.

In summary, our current study constitutes a biochemical and protein expression characterization of HATL5 in healthy and cancerous tissues, and is a first step towards deciphering the functions of this HAT/DESC subfamily member.

## Supporting Information

Table S1 Analysis of the expression of TMPRSS11b, encoding HATL5, in four gene expression array studies of human carcinomas using the Oncomine database.
(DOCX)
