# Supplementary material for: HATL5: A Cell Surface Serine Protease Differentially Expressed in Epithelial Cancers
Source: PLoS One. 2014 Feb 3;9(2):e87675. doi: 10.1371/journal.pone.0087675 (PMC3912027; doi:10.1371/journal.pone.0087675)
Supplement: Table S1 — Analysis of the expression of TMPRSS11b, encoding HATL5, in four gene expression array studies of human carcinomas using the Oncomine database. (DOCX) [file pone.0087675.s001.docx]

Table S1

| **Study ID** | **Type of Tissue Analyzed** | **P- Value** | **References** |
| --- | --- | --- | --- |
| 1 | Esophageal Adenocarcinoma vs. Normal | 1.06E-23 | Kim et al., 2010 |
| 2 | Tongue Squamous Cell Carcinoma vs. Normal | 1.21E-10 | Ye et al., 2008 |
| 3 | Floor of the Mouth Carcinoma vs. Normal | 3.28E-05 | Pyeon et al., 2007 |
| 4 | Cervical Cancer vs. Normal | 1.67E-10 | Pyeon et al., 2007 |

Kim SM, Park YY, Park ES, et al. Prognostic biomarkers for esophageal adenocarcinoma identified by analysis of tumor transcriptome. PLoS ONE. 2010;5(11):e15074.

Ye H, Yu T, Temam S, et al. Transcriptomic dissection of tongue squamous cell carcinoma. BMC Genomics. 2008;9:69.

Pyeon D, Newton MA, Lambert PF, et al. Fundamental differences in cell cycle deregulation in human papillomavirus-positive and human papillomavirus-negative head/neck and cervical cancers. Cancer Res. 2007;67(10):4605-19.
